# Informing Future Paralympic Media Approaches: The Perspective of Canadian Paralympic Athletes

**DOI:** 10.1177/21674795221103410

**Published:** 2022-05-30

**Authors:** Erin Pearson, Laura Misener

**Affiliations:** 1 6221 Western University, London, ON, Canada

**Keywords:** Paralympic, disability, media representation, photo-elicitation, Foucault

## Abstract

Media coverage of the Paralympic Games can affect how athletes with impairment and disability sport are perceived by the public. Researchers investigating media representations of disability sport have focused on how Paralympic athletes and disability sport are represented by the media. Limited research, however, has examined how Paralympic athletes perceive these representations of themselves and the meanings they attribute to such representations. The purpose of this study was to examine how Paralympic athletes make meaning of discourses of disability within Paralympic coverage. This involved semi-structured photo-elicitation interviews with eight Canadian Paralympic athletes. A reflexive thematic analysis (RTA) was used to analyze the data utilizing Foucault’s notions of discourse, power, and technologies of the self. The findings demonstrate that Paralympic athletes made meaning of the discourses of disability within Paralympic media coverage by drawing on their lived and media experiences. Athletes with more media experience articulated problematizations of dominant discourses of disability in Paralympic media coverage and engagement in technologies of the self. Knowledge generated from this study offers media personnel an informed understanding of how Paralympic athletes understand representations of disability and disability sport. This knowledge may offer insight and inform future media approaches of disability sport and the Paralympic Games.

## Introduction

The Paralympic Games are a mega sporting event for elite athletes with impairment. Media coverage of the Paralympic Games has a role in how elite athletes with impairment and disability sport are portrayed and perceived by the public (Hellwege & Hallmann, 2019; Rees et al., 2019). Historically, media representations of disability have been primarily negative, where disability is portrayed as a medical issue that individuals must “overcome” to achieve “normalcy” (Beacom et al., 2016). Most often these negative portrayals of disability showcase stereotypes where individuals are, for example, represented as victims, dependents, less than human (i.e., villains or freaks), exotic, or as superhumans for those that can “overcome” their impairments to achieve success (Cherney & Lindemann, 2019; Ellis, 2008). This is the dominant way disability has been represented by the media and is ubiquitous in Paralympic media coverage (Rees et al., 2019). Given the history of stereotypical media representations of disability and its dominance within Paralympic media coverage, this is concerning for the everyday lived experience for those living with impairment and for affecting public attitudes toward disability (Goodley, 2011). For example, the World Health Organization (2011) highlighted how the lived experiences of people with disabilities are linked to negative attitudes about disability which can affect a person’s participation in everyday aspects of life (i.e., education, employment, recreation, etc.). It is, therefore, critical that individuals with impairment are represented by the media in non-stereotypical ways.

Researchers investigating media representations of disability sport have focused their attention on examining how Paralympic athletes and disability sport are represented by the media (e.g., Beacom et al., 2016; M. Hardin & Hardin, 2005; Rees et al., 2018). The emancipatory nature of this research has led scholars to argue that medicalized discourses of disability that dominate media coverage disempower athletes by framing disability as a problem that requires “overcoming” to achieve success (Cherney & Lindemann, 2019; Rees et al., 2019). Limited research, however, has investigated how Paralympic athletes perceive representations of themselves or of others, and the meanings they attribute to such representations. Addressing this gap in research is critical because it means that the voice of those with lived experience of impairment have historically been absent from research. This absence has been a major concern in the field of disability studies (Ashby, 2011). For example, the slogan “nothing about us without us” coined by disability activist James Charlton describes how no decision (i.e., policy, research, etc.) should be decided without the full and direct participation of members of the group affected by that decision (Charlton, 1998). To understand how media coverage may be a force for positive social change regarding disability, it is therefore essential to include those with lived experience in the conversation to understand their perceptions of media representations of disability.

In this article, we examine how Paralympic athletes make meaning of the discourses of disability within Paralympic media coverage. We used Foucault’s notions of discourse, power, and technologies of the self to consider how Paralympic athletes make meaning of the discourses in the way they are portrayed in media, through engagement (or non-engagement) with technologies of the self. This enabled us to consider Paralympic athletes’ understandings of the relations of power between themselves and media. Knowledge generated from this research offers media personnel an informed understanding of how Paralympic athletes understand representations of disability and disability sport. This subsequently may offer insight and inform future media approaches of disability sport and the Paralympic Games.

## Literature Review

### Media Representations of Paralympic Athletes

Medicalized discourses of disability in Paralympic media coverage are evident of the historical origins of the Paralympic Games. The formation of the Paralympic Games by able-bodied medical professionals and sport administrators has had long lasting effects on the way Paralympic sport is covered by the media (see Howe, 2008a for more on the history of the Paralympic Games). Medicalized discourses of disability reinforce ableist norms as researchers have found evident in the language, visual images, and location of content media personnel have used to depict Paralympic athletes (e.g., Pearson & Misener, 2019; Silva & Howe, 2012; Tynedal & Wolbring, 2013).

Paralympic athletes who are represented by the media are most often portrayed as a supercrip (Cherney & Lindemann, 2019; Pullen et al., 2018; Silva & Howe, 2012). The supercrip narrative reinforces medicalized discourses of disability by framing disability as an individual problem that a person must “overcome” to achieve success (Silva & Howe, 2012). Marketing campaigns over the last decade have focused on representing athletes as “superhumans” to sell the games to the broader audience. For example, Beijing’s Superatleta campaign in 2008, the United Kingdom’s Meet the Superhumans campaign in 2012 and We’re the Superhumans in 2016, and Canada’s #Paratough Campaign in 2016. The reinforcement of ableism and downplaying of the bodily experience of impairment for Paralympic athletes misrepresents and further distances the experience of athletes from the everyday lived experiences of those with impairment (Silva & Howe, 2012). It marginalizes those in society who do not have the capacity, resources, or desire to achieve “superhuman” status (Cherney & Lindemann, 2019; Silva & Howe, 2012).

Researchers have found that a hierarchy exists in representations of disability and extends to an individual’s impairment, sport, and gender reinforcing ableist norms (DePauw, 1997). This is evident by media personnel’s comparisons of Paralympic athletes and able-bodied athletes, fixation on athletes who use technologies, and exclusion of impairment from visual representation (e.g., Bruce, 2014; Howe, 2011; Pearson & Misener, 2019). For example, in visual representations of Paralympic athletes, ableism is commonly reinforced by only showing athletes in passive positions (e.g., on the sidelines), favoring images of athletes who use technologies (e.g., wheelchairs or prosthetics), and whose impairment is invisible from representation (Bruce, 2014; Crow, 2014; Pearson & Misener, 2019). Paralympic media coverage has therefore been dominated by a single perception of the impaired body (Quinn & Yoshida, 2016). This singular type of representation does not reflect the diversity of the Paralympic community as female athletes, athletes of lesser-known sports, and athletes who have higher support needs have historically received less coverage.

### Media Personnel and Paralympic Media Coverage

Media personnel’s tendency to reflect a hierarchy of disability highlights an effort to provide audiences with palatable, ableist understandings of disability sport and the body (Purdue & Howe, 2012). These ableist types of representation may be reflective of media personnel’s struggle to understand dominant discourses of disability when covering Paralympic sport (Howe, 2008b; Purdue & Howe, 2012). For example, researchers found that most media personnel responsible for covering Paralympic Games have had little to no experience with disability or knowledge of disability sport (Howe, 2008b; Misener, 2013). Scholars have found that journalists attribute a lower status to covering Paralympic sport by repeatedly framing the Paralympics as unimportant, lesser, and not “true” sporting competition (Golden, 2003; Thomas & Smith, 2009).

Since the 2012 Paralympic Games in London, broadcasting networks such as Channel 4 (C4) in the United Kingdom and the Canadian Broadcasting Company (CBC) in Canada have, however, begun to recognize and work toward improving Paralympic media coverage (Beacom et al., 2016; CBC Media Centre, 2017; Pullen et al., 2018). This has primarily been achieved by voicing a dedication to providing not only greater media coverage, but by providing empowering coverage that focuses on athleticism first and foremost. Scholars have observed an improvement in the amount and value the media has placed on covering Paralympic sport by predominantly focusing on athletic achievement in coverage (e.g., Maika & Danylchuk, 2016; Pearson & Misener, 2019; Pullen et al., 2018). For example, researchers investigating Canadian media representations of the 2012 and 2016 Paralympic Games found that Canadian media companies have primarily represented Paralympic athletes as athletes (Maika & Danylchuk, 2016; Pearson & Misener, 2019).

Despite the positive shift, medicalized discourses of disability have remained prevalent in media coverage as media personnel have focused on the supercrip, overcoming, and comparison narratives depicting the participation of a Paralympic athlete at the Paralympic Games. This tension, demonstrated by media companies voicing a dedication to producing empowering coverage yet depicting Paralympic athletes using medicalized discourses of disability, can be understood as the Paralympic Paradox (see Purdue & Howe, 2012).

### Paralympic Athletes’ Perspectives of Media Coverage

The aforementioned literature demonstrates the complex and multiple discourses Paralympic athletes are confronted by in Paralympic media. The literature highlights the tensions experienced by the media and Paralympic stakeholders who are trying to navigate the complexities of disability sport coverage. Limited research, however, has included the voices of Paralympic athletes who are represented in media and their perspectives of Paralympic media coverage. Researchers have primarily used content analysis (e.g., Beacom et al., 2016; Chang et al., 2011; Tynedal & Wolbring, 2013), and frames analysis (e.g., Maika & Danylchuk, 2016; Misener, 2013; Pearson & Misener, 2019) to examine Paralympic media coverage. The few studies that have investigated athletes with impairments perspectives of disability sport media coverage found that participants were unhappy with the overall lack of disability media coverage and content that highlighted medicalized discourses of disability (e.g., Brittain, 2004; M. Hardin & Hardin, 2005; Hargreaves & Hardin, 2009).

In addition, few scholars have specifically focused on the perspectives of Paralympic athletes’ understandings of Paralympic media coverage. For example, the work of Howe (2008b, 2011) and Peers (2009, 2012) are important as they provide perspective regarding media coverage and disability sport as both scholars and retired Paralympians. Howe (2008b, 2011) and Peers (2009, 2012) have tended to reject the use of dominant discourses within media coverage. For example, by arguing how medicalized discourses of disability homogenize disability experiences resulting in the loss of an opportunity for media to “acknowledge the pervasiveness of difference” (Silva & Howe, 2012, p. 191). Peers (2012) further called on members of the disability sport community to stop reproducing and accepting dominant discourses of disability and for members to begin participating “in de-composing the stories, cultures and industries that disable” (p. 186). Furthermore, other scholars have recently begun to include Brazillian Paralympians’ perspectives of media coverage in their research either as the primary foci of study (see Marques et al., 2014), or as one part, in combination with other factors, that may shape a Parlympian’s narrative identity (see Kirakosyan, 2021). This recent development in the literature marks a notable shift toward including the voices of Paralympic athletes in research on Paralympic coverage, but largely athletes’ perspectives remain absent overall and the perspectives of athletes from other cultural contexts.

As subjects of the media narratives, Paralympic athletes should have a voice in the ways in which these discourses are framed. Thus, our work focuses on how Canadian Paralympic athletes make meaning of discourses of disability in Canadian Paralympic media coverage. We seek to contribute to the body of literature by examining Canadian Paralympic athletes’ perspectives of coverage beyond their general likes/dislikes to contribute an informed understanding of an athlete’s media experience situated within the sociohistorical discourses of disability, and power relations with media.

### Theoretical Framework

In this study, we draw on Foucault’s notions of discourse, power, and technologies of the self to guide our understanding of how Paralympic athletes make meaning of the discourses of disability within coverage through engagement (or non-engagement) with technologies of the self. Michel Foucault’s reconceptualization of power has had a significant impact in the fields of social sciences and humanities, and within sports research (e.g., Hardin, 2011; Markula, 2003, 2004; Markula & Pringle, 2006; Spowart et al., 2010; Thorpe, 2008). Most contemporary understandings of power view power as a repressive force that only certain people possess. Foucault (1978) alternatively argued that power is everywhere as a series of omnipresent, ever-shifting “multiplicity of force relations” that exist at all levels of social relationships, albeit often in unequal ways (p. 93). In his work, *The Subject and Power* (1982), he explained that the objective of his work over the years has “not been to analyze the phenomena of power,” rather it has been to “create a history of the different modes by which, in our culture, human beings are made subjects” (p. 777). He proposed three modes of objectification which transform individuals into subjects: (a) scientific classification, (b) dividing practices, and (c) subjectification.

The first two modes are focused on how individuals are disciplined, classified, and normalized by social processes that they have little control over. The first mode of objectification, scientific classification, was concerned with how people come to recognize themselves as objects and subjects of scientific knowledge (Foucault, 1982). Foucault (1970) proposed that the human sciences facilitated to construct universal classifications of individuals, and in this process, individuals become objectified. In the context of disability, an example of scientific classification can be demonstrated in the use of medical examinations and “truths” (i.e., that is, the types of discourse society accept and makes function as true) to declare someone as disabled (Foucault, 1980). The second mode of objectification, dividing practices, Foucault (1982) described how the subject is either “divided inside himself or divided from others” (p. 778). Here, individuals may further be sub-categorized and divided from each other through a series of “social and spatial divisions” based on their degree of normalization (Markula & Pringle, 2006, p. 26). This is represented in the process of classification in parasport where medical personnel group athletes with disabilities into different hierarchal numerical classes based on their degree of activity limitation resulting from impairment. We also see this demonstrated in media coverage of Paralympic athletes where media personnel represent Paralympic athletes using stereotypical discourses of disability such as a supercrip, victim, cyborg, and/or in direct comparison to able-bodied, Olympic athletes (e.g., Olympians 2.0).

The third mode of subjectification, however, was the prime way Foucault understood possibilities for creating oneself as an ethical subject within power relations. What is notable about the third mode is that it marks a shift in his work to examine the processes in which a person is active in self-formation. Foucault (1988) identified those processes as technologies of the self, which he described to, “permit individuals to effect by their own means or with the help of others a certain number of operations on their own bodies and souls, thoughts, conduct, and way of being” (p. 18). The purpose of this, Foucault (1988) described, was so that individuals may transform themselves within relations of power, “to attain a certain state of happiness, purity, wisdom, perfection, or immortality” (p. 18). In the context of media, an example of this may be how Paralympic athletes use their media training during conversations with media personnel to articulate their preferred discourse (e.g., language to describe disability or impairment), or possibly a reverse discourse (e.g., athlete first rather than stereotypical discourses), to be used in representation. According to Foucault (1978), reverse discourses use the same or similar vocabulary as a dominant discourse but produce an opposing strategy or social effect. For example, Thorpe (2008) examined female snowboarder’s understandings of dominant discourses of femininity in snowboarding media representation and found that some female snowboarders drew on reverse discourses to negate the effect of overly sexualized images in snowboarding media.

Although the technologies of the self can act as practices of freedom (i.e., practices an individual may use to transform themself within power relations), certain conditions apply. According to Foucault (1988), the first step of technologies of the self involves an individual gaining the ability to problematize their identity and the discourses that govern him/her within relations of power. Only after such reflection could a person engage in active work on the self to develop or achieve this form (i.e., ethical work). Foucault (2000b) argued that “ethics is the considered form freedom takes when it is informed by reflection” (p. 284). Foucault developed the notion of problematization and proposed that ethical self-creation required problematization, a style of thought in which:

Thought is freedom in relation to what one does, the emotion by which one detaches oneself from it, establishes it as an object, and reflects on it as a problem … for a domain of action, a behaviour, to enter the field of thought, it is necessary for a certain number of factors to have made it uncertain, to have made it lose its familiarity, or to have provoked a certain number of difficulties around it. (Foucault, 2000a, p. 117)

The first part of the quote emphasizes problematizations as process of explicit, critical thought, and the second part of the quote describes how this thought process is contextual. For example, an individual might be prompted to problematize an aspect of their sporting experience due to a traumatic event and/or through engagement in other games of truth (Crocket, 2014; Pringle & Hickey, 2010). Much of Foucault’s (1984) latter thinking about problematization was about positioning radical critique, or “the critical work that thought brings to bear on itself,” as the main task of a contemporary philosopher (p. 9). It is, therefore, important to remember that individuals may not express problematizations using critical sociological and pedagogical scholarship but may express problematizations on their own terms (see Crocket, 2016). We address this consideration in our method’s section.

Drawing on Foucault’s work can thereby support our examination of how Paralympic athletes make meaning of the discourses of disability within coverage through engagement (or non-engagement) with technologies of the self. Foucault’s work supports our microanalysis of Paralympic athletes’ problematizations and their use of technologies of the self within the sociohistorical discourses of disability, and power relations with media.

## Method

Given our intent to offer space for Paralympic athletes to discuss their perceptions of the discourses present in the media, we employed photo-elicitation methods. Photo-elicitation interviews (PEIs) can help invoke deeper conversations with participants about their perceptions of their own Paralympic media representation. We utilized researcher-driven PEIs following a semi-structured interview format with the option for participants to include their own text/visuals from media coverage in the interview. This allowed for participants to have a choice in the data discussed during their interviews and to help understand participants’ perspectives (Hatten et al., 2013). This method also supports a critique made by Crocket (2016) regarding how previous empirical research that has investigated problematizations of individuals in sport has failed to consider participants’ problematizations on their own terms. Our choice of using photo-elicitation methods helped us address this consideration by providing athletes the agency to include their own text/visuals to use during the interview as a point of reflection thus creating space for athletes’ potential problematizations on their own terms.

### Participants

Paralympic athletes eligible for participation in this study had been featured in at least three online articles from Canadian national media outlets from the 2012 Paralympic Games and onward. This included Paralympic athletes who had competed at the 2012 Paralympic Games up until the Winter Paralympic Games in 2018. The 2012 Paralympic Games were chosen as the starting point for athlete recruitment based off the notable increase in interest and shift in approach for covering the Paralympic Games (Beacom et al., 2016; CBC Media Centre, 2017; Pullen et al., 2018). Participants were recruited for this study via their national sport governing bodies where the request to participate was distributed to the athlete by a coach and/or high-performance coordinator. Paralympic athletes who were interested in participating in the study were asked to contact the researcher directly to schedule an interview.

The result of this recruitment process was a sample of eight Paralympic athletes (four women and four men). For reasons of confidentiality, we have not provided a table of participant demographics as the Canadian Paralympic sport community is small, and participants could easily be identified if this information were presented. The sample, however, was diverse in terms of age, impairment, sport, and number of Paralympic Games. The sample included a mix of both summer and winter Paralympic athletes and both individual and team sports. Six out of the eight participants competed in different sports. All participants had a least one Paralympic Games experience from 2012 through to 2018 with some athletes having additional Paralympic Games experience prior to the 2012 Games. Pseudonyms were assigned to each participant at the time of transcription of the interview.

### Data Collection

The portfolios we created for our participants included a minimum of three online media articles (text/visuals) published within our selected timeframe (2012 onward) and were written about the Paralympic athlete from one or more of the three major national media companies in Canada: (a) CBC, (b) the Globe and Mail, and (c) the National Post’s online webpages. All three sources are Canadian media companies providing Canadians with national Paralympic media coverage.

To create the participants portfolios, we began with an initial search of the three media companies’ online databases. This resulted in a total of *n* = 184 articles found across all participants. All articles were next reviewed and articles not relevant to the study were removed. The articles considered not relevant were articles outside of the timeframe, did not prominently feature the athlete in the article (e.g., their name was mentioned in passing), and/or were videos or audio clips with no text. In total, this process resulted in *n* = 94 articles. Next, we employed a deductive coding analysis utilizing Paralympic media representation research by Pearson and Misener (2019) who analyzed dominant mediated discourses of disability in Canadian Paralympic media coverage. Pearson and Misener (2019) found that the dominant frames used by media to depict Paralympic athletes for the 2016 Paralympic Games were: (a) the athletic frame (e.g., represented as an athlete first), (b) the stereotypical frame (e.g., the supercrip, overcoming and comparison narratives), (c) the informative frame (e.g., educational and players own voice pieces), and (d) the multi-dimensional frame (e.g., focus on roles with family, friends, and life outside of sport). After the coding process was complete, at least three coded articles (text/visuals) were selected for each participant’s specific media portfolio.

The decision to create coded portfolios for this study, and not include all articles found in the search criteria, was chosen for two reasons: (a) to present the participants with a portfolio that was reflective of the multiple discourses that Paralympic athletes are confronted by within their representation, and (b) to present a portfolio in a length that participants were able to reasonably engage with and reflect on before and during the interview process. For example, some participants’ portfolios may have ranged from 33 articles while others at only five articles in length. Designing the portfolios to be three to six coded articles in length per athlete resulted in a presentation that was reflective of their overall Canadian media representation, but also respectful of the athlete’s choice to participate in this study during their demanding schedules as high-performance athletes. This also ensured consistency in the use of portfolios across the interview process and allowed PEIs to be completed within 60–90 minutes in length. Table 1 provides a summary of the search and creation process for the participants’ portfolios. Table 2 provides a summary of the breakdown of the coded articles used within each participant’s portfolio.

**Table 1.** Summary of Search and Creation Process for Participant Portfolios.

Name	Total # of Initial Search Articles	Total # of Coded Articles	Athletic	Stereotypical	Informative	Multi-Dimensional
Trent	30	14	8	6		
Rafael	10	5	2	3		
Tricia	19	15	4	10	1	
Lance	21	8	2	4	2	
Janice	7	6	3	1	2	
Michelle	72	33	19	11		3
Carlo	14	8	7		1	
Caroline	14	5	2	1	1

**Table 2.** Summary of Participant Portfolios.

Name	Total # of Articles	Athletic	Stereotypical	Informative	Multi-Dimensional
Trent	6	3	3		
Rafael	3	1	2		
Tricia	4	1	2	1	
Lance	4	1	2	1	
Janice	4	1	1	2	
Michelle	5	2	2		1
Carlo	4	3		1	
Caroline	3	1	1	1	

Once the portfolios were created, the portfolios were emailed to the participants a week prior to their PEI. This step allowed the participant time for reflection prior to the interview and the option to select additional text/visuals to be included. All participants were reminded of this option during the PEI and that they may email the researcher post interview with any additional text/visuals they would like to include. The total number of articles selected by the researchers for all participant portfolios was *n* = 33 texts which included *n* = 58 visual images within the articles. One article (e.g., one text and three visual images) was sent to the researcher to be included in the participant’s portfolio during their interview. The total number of articles including the articles sent to the researcher was *n* = 34 texts and *n* = 61 visual images.

A single, PEI was completed with each participant via Zoom Web Conferencing. Interviews followed a semi-structured format with the first author guiding the conversation about how each athlete felt about their own representations, general discussion about Paralympic coverage, and desired directions for future coverage. While the portfolio focused on their own media coverage, athletes were also encouraged to discuss their broader perceptions of Paralympic discourses present in the media. All participants acknowledged that they felt the portfolio created for them represented their media presence and no participants sent any additional text/visuals to be discussed post interview.

### Data Analysis

A reflexive thematic analysis (RTA) was used to analyze the data (Braun et al., 2016; Braun & Clarke, 2019). Reflexive thematic analysis was chosen for this study because it is a flexible method which allowed the researchers to analyze the data both inductively and deductively (i.e., theoretically) at times while also considering the researcher’s own position within the study (i.e., reflexively) (see Braun & Clarke, 2019 for more information on RTA). Data analysis was therefore not only based on the verbal and visual data set, but also guided by Foucault’s notions of discourse, power, and technologies of the self. This method of analysis allowed the researchers to work back and forth through Braun et al.’s (2016) six phases of conducting thematic analysis recursively (see Braun et al., 2016 for a description of the six phases). Where there was any discrepancy between the researchers in terms of generating codes or defining the themes, they met to discuss returning to Foucault’s notions of discourse, power, and technologies of the self as a source of guidance to find common ground for any disagreements. The process proved extremely challenging as the media experiences of Paralympic athletes are complex, nuanced, and fluid. This challenged the researchers to develop themes that felt representative of the complexity. Ultimately, the researchers agreed upon two key themes that are presented below.

## Findings and Discussion

The two overarching themes developed to represent the findings are: (a) (mis)understandings of discourses of disability, and (b) power relations with media. The first theme, “(mis)understandings of dominant discourses of disability,” describes how Paralympic athletes made meaning of the dominant discourses of disability in Paralympic media coverage. The second theme, “power relations with media,” describes the Paralympic athletes’ interactions with media personnel and the relations of power encompassing being represented in Paralympic media coverage.

### (Mis)understanding of Discourses of Disability

The findings of this study demonstrate that the media experience of a Paralympic athlete is complex. Each media experience was unique as each Paralympic athlete was confronted with multiple and contradictory discourses depending on their impairment, sport, and gender. Paralympic athletes made meaning of the discourses of disability depicted in Paralympic media coverage by drawing on their lived (e.g., education, gender, impairment, sport, number of games competed at) and media experiences (e.g., number of media interactions, perception of consuming media, perception of media experiences). While all interviewees drew on both their lived and media experience to make meaning of discourses of disability, the amount of media experience an athlete had was the only commonality between athletes who demonstrated problematizations of discourses from those who did not. This was consistent across participants no matter their age, gender, sport, impairment, or whether their impairment was acquired or congenital.

Interviewees with less media experience were less likely to express conscious problematizations of discourses of disability. These athletes simply expressed a level of platitude in terms of their perspective of some discourses used in Paralympic media coverage without much critical self-reflection to support their understanding. However, they also had contradictory perspectives of how they ought to be presented. For example, two athletes who competed at one Paralympic Games spoke about how they enjoyed supercrip narratives within media advertising campaigns but later expressed that they would like to be represented for their sporting prowess first and foremost, and not the pity narratives. Tricia, who has media experience from one Paralympic Games, initially expressed enjoyment of media employing the supercrip narrative but later noted that she did not want to only be represented as someone whose athletic success was because of her ability to “overcome” her disability. She wants media personnel to focus on the athletic “accomplishments that you’ve made and how hard you had to work to get there.” By doing so, these athletes accepted some discourses of disability in media coverage and rejected others. They stated that they had only positive experiences with media personnel and of consuming Paralympic media coverage. Markula (2004) suggested in her study of exercise instructors, if an individual did not have cause to find their experiences problematic, they may not have been exposed to discourses and power relations yet that could trigger or facilitate a process of problematization. Athletes with less media experience may not have encountered occurrences where representation or interactions with media personnel were problematic, thus remained relatively positive about media coverage.

Interviewees that expressed conscious problematizations of discourses of disability in Paralympic media coverage were athletes that had the most media experience. Athletes with greater media experience had multiple years of media experience spanning several Paralympic Games, and/or had multiple media experiences related to success at a Paralympic Games. For example, Michelle had several years of media experience competing at multiple Paralympic Games. She provided a timeline of how her media experience has improved over the last 5 years drawing on examples in her portfolio. In the first article from 2012 we discussed, she explained how she did not like that the journalist focused on the origin story of her impairment for the entire article. She further highlighted on the positive improvements she has seen with her representation today as media personnel now primarily focus on her athletic success and abilities highlighting her as an athlete with a disability. Carlo, who had multiple Paralympic Games experiences, additionally highlighted an observable shift over the past few decades toward positive media experiences. He reflected on the use of superhuman language in his coverage and comparison narratives between able-bodied and Paralympic athletes. These reflections (or the inclusion of their own text/visuals) did not occur in our interviews with Paralympic athletes with less media experience. This finding supports Thorpe’s (2008) study which noted that more experienced female snowboarders were the ones who were more likely to develop a critical awareness of dominant discourses of sexism in media. Thorpe (2008) argued that:

The more snowboarding experience and cultural knowledge an individual has, the more likely he or she is to develop the ability to weigh up the competing versions of femininity on offer in the snowboarding culture and problematize some of these [discourses]. (pp. 214–215)

The more media experience a Paralympic athlete therefore had, the more likely they were to observe inconsistences between their lived experience and the mediated discursive constructions of disability.

Some interviewees drew on reverse discourses to demonstrate the inconsistences they perceived between mediated discursive constructions of disability and their lived experience. These athletes discussed wanting to be represented by the media based on their sporting prowess calling for proportionate media representation. For example, Carlo stated:

I view the word inspiration as sort of a dirty word because to me it represented how people looked at me and didn't really care what kind of athlete I was or didn't really see me as an athlete. They just saw me as symbolic as somebody who's just like a champion for getting out of bed in the morning.

Furthermore, Janice explained how “I would never say I’m superhuman by any means. Just someone who loves to play sports and has a good time with it. That doesn’t make me a superhuman.” These experienced Paralympic athletes rejected dominant discourses of disability such as the supercrip narrative and discourses that attribute sporting success to “overcoming” disability. These Paralympic athletes emphasized the need to move away from medicalized and ableist discourses of disability (e.g., Beacom et al., 2016; Pearson & Misener, 2019; Pullen et al., 2018; Silva & Howe, 2012; Tynedal & Wolbring, 2013).

Interviewees who drew on reverse discourses also rejected dominant discursive constructions of disability and sport by expressing their desire for media to focus attention on their sporting proficiency. Reflecting on DePauw’s (1997) construction of sporting bodies, athletes highlighted how sport remains an able-bodied, masculinized space. Thus, representations that highlighted their sporting prowess should not erase disability or their intersectionality. Rather, athletes expressed the desire for media’s disruptive potential. For example, Rafael stated he likes articles that have a “good balance between [his] story and the [event] or the training itself.” Interviewees felt that the inclusion of their impairment is what makes their story unique and that their impairment is an important part of their identity. Interviewees stated that articles that focused only on the results and athletic achievements were less enjoyable to read than articles that included both a Paralympic athlete’s athletic achievements and backstory. The story offers space to disrupt dominant understandings of sporting practices.

Furthermore, some athletes also discussed that it was not the inclusion of their impairment that necessarily mattered in the media coverage, but the language used to describe their impairment. Janice discussed her frustration with media:

I have no problem talking a little bit about how I got started in the sport or whatever. I just don't want that to be the primary focus of the article. I don't want it to be focused on my disability. I want it to be focused on my ability.

The subtle shift in the ableist narratives is critical. All Paralympic athletes expressed concerns about the constant comparison to able-bodied athletes. Michelle argued that she felt devalued because her sporting excellence was secondary to her able-bodied counterparts: “why isn’t [able-bodied athlete name] me 2.0, why am I [able-bodied athlete name] 2.0. I’m not [able-bodied athlete name] 2.0 thanks.” This discourse is reflective of the failure of media personnel to view the impaired sporting body as legitimate and as valuable as able-bodied athletes.

Finally, interviewees recommended that journalists who are covering Paralympic sport be educated in the type of language they use to describe disability. Their own media experiences highlighted the “discomfort” media personnel have with disability. Interviewees felt they were able to help focus the narratives and support the directions of the storylines to better reflect sporting successes and disability. Caroline suggested that “individuals might not feel comfortable sharing their story or might not want it represented a specific way. [Media] should be giving choice, and voice is therefore really important in their narrative as well.”

### Power Relations with Media

Paralympic athletes’ reflections of power relations with media demonstrates how media personnel’s use of dominant discourses of disability (in texts and visuals) affect what bodies are believed to belong in sport. Some interviewees reflected on how the language media personnel choose to depict Paralympic athletes still reinforces dominant understandings of disability. Scholars have noted how this may affect public perceptions of Paralympic athletes and disability sport. The athletes discussed how media campaigns that they have observed or been involved in tend to reinforce the stereotypical understandings of ability. Caroline explained:

Athletes or people with disability are portrayed and forced into particular categories within media. One’s the superhuman and one’s a victim...it reinforces [negative] ideas for a lot of people. Just in general, when you tell them that you’re involved with Paralympic sport, their comments go along the lines that Paralympic athletes work harder or that it’s more impressive that you’re in the Paralympics. Which to me isn’t something that is necessarily true. So just kind of one of those stereotypes that maybe this campaign, even by the name of it, would be perpetuating.

Furthermore, many athletes noted how action shots were their preferred type of visual representation as this reinforced how they felt about their bodies and their desire to be celebrated for their sporting prowess. For example, Trent reflected on the action shots of himself depicted in his portfolio as “a very accurate representation of [his] sport,” displaying the physicality of himself and his teammates. This supports previous findings that argued against media representing Paralympic athletes through a participation lens by choosing to feature athletes, particularly female athletes, in primarily passive positions (Crow, 2014; McPherson et al., 2016; Pearson & Misener, 2019; Quinn & Yoshida, 2016).

Interviewee’s reflections also demonstrated how media personnel’s perceptions of disability affect the experience of Paralympic athletes. All interviewees reflected on how their perspective of Paralympic media coverage has changed over the past decade from a negative to a mostly positive perception. A positive experience was described as working with media personnel who were prepared, knowledgeable about the athlete’s history, and treated them as an athlete. The athletes noted that media who understand disability in terms of dominant discourses exhibited a lower expectation and overall value of covering disability sport compared to able-bodied sport. This was reflected by interviewees providing examples of media personnel being unprepared for interviews and/or who chose images that depict athletes based on dominant discourses of disability. For example, Michelle expressed her frustration of being interviewed by media personnel who did not understand her impairment or sport. She stated, “you can tell in the first minute. Reporters ask me how I don’t [move] in circles…so I know right away that they have no clue.” Their insights highlighted a key concern and supports previous findings that media who cover Paralympic sport are not adequately trained or educated, demonstrating the low value placed on these Games (Howe, 2008b; Misener, 2013).

While interviewees often had difficulty reflecting on their own experiences with media, they discussed this in relation to the low quantity and overall lack of diversity of Paralympic media coverage. All athletes suggested that proportionate representation both in terms of the quantity and quality of representation was necessary for them to gain legitimacy. They discussed the importance of language that represents them as athletes, the need to use diverse mediums to broadcast disability sport, representing more sport events than just the Paralympic Games, and providing more in-depth and critical coverage comparative to able-bodied sport. For example, interviewees described how they enjoyed when media personnel shift away from the simple introduction type of article. Trent, for example, noted this type of article from his own portfolio which describes “what is [your sport] and tell us about yourself.” Carlo called this pattern of articles “the cycle of re-introducing the media” in which “every four years it was like, hey, who are you again?” Interviewees felt the cycle of re-introduction perpetuated the problematic discourses of focusing on the impairment story, the overcoming, and never truly reflecting the diversity or depth of the Paralympic athlete experience.

Finally, some Paralympic athletes did demonstrate a strategic move toward self-determined technologies of the self by utilizing their media training and lived experience as a tool to control conversations with media personnel. They actively sought to disrupt the power dynamic that has perpetuated the problematic stereotypes. A few athletes described how they actively drew on reverse discourses during their interviews with media to resist media personnel’s dominant understandings disability. For example, Janice argued:

As athletes, you have a voice, and you have an opportunity to be covered the way you want to be covered. It’s up to you to guide that as well. So, if you’re not happy sometimes with the way it’s being covered, maybe you need to reflect on the answers you give in the interview or even your approach to the interview.

This sentiment shifts the power potential back to athletes but places a lot of onuses on Paralympic athletes, who may not have that experience to offer this voice. The more experienced athletes further expressed how they had learned to engage in reverse discourses to educate the media and public about the legitimacy of Paralympic sport. Lance described this in relation to his own media experience by stating:

The people that I’ve met and some people you could see in the beginning they might be and just not super interested. Then it’s the athlete’s responsibility as well to teach them a little bit and get them interested.

He highlighted articles from the portfolio that demonstrated how he used the opportunity of the media interview to “put a fun spin on” questions to help highlight messaging about the sport and not an overcoming narrative. As Janice highlighted, using the opportunity of the interview to promote the actual sport offers a potential to shift the conversation. Paralympic athletes’ efforts to engage in reverse discourses used by media personnel demonstrate a point of resistance for athletes within power relations with media personnel. These athletes demonstrated the capacity to discuss with media personnel their preferred type of representation, albeit the relations of power are highlighted by the ways in which media report the story. Noting a particular article from her own portfolio where she seemed to try to change the conversation, Janice explained:

I’m always big on trying to redirect it into the sport, athleticism, and the accomplishment versus the overcoming, you know, the inspirational message. Nothing like being an inspiration to someone, but at the end of the day, the journalist writes what they want to write, right? You can give the best of the best and it’s working with them to move beyond that message. But when they leave us and they have the recording and they go back and they write their article, it is what it is.

Athletes expressed the need to resist the dominant understandings that objectify the “disabled athlete,” but ultimately believed the power continues to lie with the media. Interviewees expressed their frustration with media personnel that precluded an athlete’s efforts to be represented for their sporting successes and thereby, limited their ability to exercise agency in the relations of power. If these power structures are to shift, media personnel will need to recognize the relations of power between themselves and Paralympic athletes. Interviewees further highlighted the need for media personnel to receive training and education regarding disability and disability sports prior to covering a disability sport event. Previous research has demonstrated that media personnel covering the Paralympic Games often have no prior experience or knowledge of disability sport (Howe, 2008b; Misener, 2013). Thus, while some athletes noted subtle acts of resistance against the dominant, negative discourses, the power structures continue to exclude their voices.

## Conclusion

The findings of this study demonstrate that Paralympic athletes make meaning of the discourses of disability within Paralympic media coverage by drawing on their lived and media experiences. Paralympic athletes who are well represented by the media are confronted by multiple and contradictory discourses of disability in coverage of themselves and of other Paralympians. This was exhibited in the portfolios constructed for this study and through Paralympic athletes’ reflections of their media experiences. Using Foucault’s notions of discourse, power, and technologies of the self, our analysis demonstrates that some Paralympic athletes expressed conscious problematizations of the discourses of disability promoted in Paralympic media coverage. Those athletes who problematized dominant discourses were those with the most media experience. Some athletes drew on reverse discourses of disability, rejecting dominant medicalized discourses such as the supercrip and comparison narratives. Some Paralympic athletes further expressed engagement in technologies of the self by using their media training and lived experience as a tool to control conversations with media personnel. In doing so, athletes worked to shift the conversation away from medicalized discourses of disability to reverse discourses that highlighted their sporting prowess. Paralympic athletes who did not demonstrate problematizations of discourses of disability articulated only positive media involvements but had limited examples from their own portfolios to highlight these experiences.

Furthermore, Paralympic athletes understood themselves as subjects within relations of power with media. Some athletes attempted to use reverse discourses during their interactions with media personnel as a form of resistance from the stereotypical discourses of disability. Athletes, however, had limited success in shifting dominant discourses of disability and controlling their representation despite their efforts to educate media personnel. Through their media experiences, athletes highlighted the importance of media personnel having education and training about the dominant discourses of disability to appropriately cover disability sport. They also highlighted that it is more than media personnel having the training to cover disability sport, but media personnel need to see the value in covering disability sport. If media personnel understand disability only in terms of medicalized discourses this negatively effects the media experience for a Paralympic athlete. The quality and quantity of Paralympic media coverage remains a problem for shifting the discourses. The Paralympic athletes we interviewed called for future representation of the disability sport that is proportionate to able-bodied sport.

This study contributes to the literature in several ways. First, our study forefronts the perspectives of Paralympic athletes in the discussion of Paralympic media coverage which has been largely absent from previous research. This contributes to the important and recent development in the literature of including athletes’ perspectives in the body of disability sport media research. Our work also contributes to the body of literature by providing an athlete perspective that goes beyond their general likes/dislikes of coverage. Our findings demonstrate an informed understanding of a Paralympic athlete’s media experience situated within the sociohistorical discourses of disability, and power relations with media. Finally, our study contributes to the growing body of sport literature that has utilized Foucault’s work of technologies of the self and through our consideration of athletes’ problematizations on their own terms (e.g., providing athletes the opportunity to include text/visuals they felt reflected their experience). This method was beneficial for our study, but it is important to keep in mind that even though some athletes in our study did not express problematizations, it does not mean that those athletes have never questioned, or critically reflected upon the discourses of disability within coverage. It also cannot be generalized that those athletes never will. Problematizing one’s identity is a dynamic process which may result in athletes problematizing discourses of disability at different moments over the course of their lives (Crocket, 2016). What we present in our analysis is an understanding of how athletes made meaning of the discourses of disability within coverage at the point in time our research was conducted. Furthermore, Markula (2004) argued that the participants who did not express conscious problematizations in her study may not have been exposed to discourses and power relations yet that could trigger or facilitate a process of problematization. Future research should, therefore, explore “the possibilities of the embodied, affective, and emotional aspects of problematization” (Crocket, 2016, p. 33). For example, using a longitudinal research approach and/or by using multiple data collection methods (e.g., interviews, observations, participants keeping journals) to build off the findings of this research and to further consider athletes’ problematizations in different ways (Crocket, 2016).

Future studies should also explore Paralympic athletes who are not represented by the media to understand their perspectives of the dominant discourses of disability in Paralympic media coverage. Their perspectives would provide significant insights into the media experience of someone who is marginalized from representation. Finally, future research should investigate the perspectives of media personnel to understand their own experiences with dominant discourses of disability in Paralympic media coverage. This is particularly relevant considering recent works that have highlighted media’s social change agenda around Paralympic sport (Pullen et al., 2018). Media personnel’s understandings of the power relationship with Paralympic athletes would help us understand how the media experience for Paralympic athletes may be improved going forward.
